# Dupilumab efficacy by disease severity in pediatric type 2 asthma

**DOI:** 10.1111/pai.70196

**Published:** 2025-11-07

**Authors:** Daniel J. Jackson, Monika Gappa, Leonard B. Bacharier, Antoine Deschildre, Kristie Ross, Changming Xia, Olivier Ledanois, Katherine Miller

**Affiliations:** ^1^ University of Wisconsin School of Medicine and Public Health Madison Wisconsin USA; ^2^ Children's Hospital Evangelisches Krankenhaus Düsseldorf Düsseldorf Germany; ^3^ Monroe Carell Jr. Children's Hospital at Vanderbilt University Medical Center Nashville Tennessee USA; ^4^ Pediatric Pulmonology and Allergy Department, Jeanne de Flandre Hospital CHU Lille, University of Lille Lille France; ^5^ UH Rainbow Babies and Children's Hospital Cleveland Ohio USA; ^6^ Regeneron Pharmaceuticals Inc. Tarrytown New York USA; ^7^ Sanofi Paris France

**Keywords:** asthma, clinical trial, disease exacerbation, dupilumab, lung volumes, patient‐reported outcome

## Abstract

**Background:**

Dupilumab demonstrated efficacy in children with asthma in phase 3 VOYAGE (NCT02948959) and open‐label extension EXCURSION (NCT03560466) studies. This post hoc analysis assessed dupilumab's long‐term efficacy by asthma severity at parent study baseline.

**Methods:**

Children (6–11 years) with moderate‐to‐severe type 2 inflammatory asthma (PS baseline blood eosinophils ≥150 cells/μL or fractional exhaled nitric oxide ≥20 ppb) from placebo‐controlled VOYAGE who enrolled in EXCURSION received dupilumab every 2 weeks for an additional 52 weeks. We assessed annualized severe exacerbation rates, change in pre‐bronchodilator percent predicted forced expiratory volume in 1 second (FEV_1_) and FEV_1_ *z*‐score, and Interviewer‐Administered 7‐item Asthma Control Questionnaire (ACQ‐7‐IA) score in subgroups with moderate (medium‐dose inhaled corticosteroid (ICS), pre‐bronchodilator percent predicted FEV_1_ ≥ 80%) and severe (high‐dose ICS, pre‐bronchodilator percent predicted FEV_1_ < 80%) asthma at parent study baseline.

**Results:**

Among 179 children (104 with moderate, 75 with severe asthma), dupilumab compared to placebo reduced unadjusted annualized severe exacerbation rates during VOYAGE in children with moderate (0.22 vs. 0.37) and severe (0.41 vs. 1.07) asthma, with sustained reductions in EXCURSION (moderate: 0.07 and 0.07; severe: 0.24 and 0.19). At VOYAGE Week 52, the change from the parent study baseline in pre‐bronchodilator percent predicted FEV_1_ was 8.6 versus 2.1 percentage points with dupilumab and placebo in children with moderate asthma, and 16.7 versus 9.8 percentage points in children with severe asthma. Improvements in the change from the parent study baseline in pre‐bronchodilator FEV_1_ *z*‐scores and ACQ‐7‐IA scores were also observed with dupilumab treatment in both subgroups.

**Conclusion:**

Long‐term dupilumab treatment reduced exacerbation rates and improved lung function and asthma control in children with type 2 asthma irrespective of disease severity, with numerically greater improvements in children with severe asthma.

**Abbreviations**
ACQ‐7‐IA: Interviewer‐Administered 7‐Item Asthma Control Questionnaire
CI: confidence interval
FeNO: fractional exhaled nitric oxide
FEV_1_
: forced expiratory volume in 1 second
ICS: inhaled corticosteroids
IgE: immunoglobulin E
IU: International Units
LS: least squares
MMRM: mixed‐effect model with repeated measures
ppb: parts per billion
Q: quartile
SD: standard deviation
SE: standard error
TARC: thymus‐ and activation‐regulated chemokine

**Key message**

Dupilumab improved clinical symptoms and asthma control in children with type 2 asthma irrespective of disease severity at baseline, with numerically greater improvements in children with severe asthma, highlighting the potential of targeted biologic therapies for optimizing outcomes in children with difficult‐to‐treat asthma.

## 1 INTRODUCTION

Asthma represents a significant health concern worldwide among children. In the United States, it affected approximately 8.1% of the pediatric population in 2016–2018.^1^ Trends show that asthma prevalence increases with age in children, from 4.0% among infants and toddlers (0–4 years) to 8.8% among school‐aged children (5–11 years) to 10.5% among adolescents (12–17 years).^1^ The underlying pathology in most asthma cases involves type 2 inflammation, predominantly orchestrated by T helper type 2 (Th2) cells secreting interleukins (IL‐4, IL‐5, and IL‐13), eosinophils, and mast cells, among other factors.^2^ Asthma with elevated markers of type 2 inflammation comprises up to 50% of mild‐to‐moderate asthma cases and likely represents an even larger proportion of patients with severe asthma,^2^ highlighting an unmet need for therapies that target type 2 inflammation in asthma.

Current guidelines distinguish between mild, moderate, and severe asthma through retrospective assessment after 2–3 months of asthma treatment of the intensity of treatment required to control symptoms and exacerbations.^3^, ^4^ Moderate asthma is characterized by symptoms that are often manageable with standard therapies such as low‐ or medium‐dose inhaled corticosteroid (ICS) and a long‐acting β_2_ agonist.^3^, ^4^ In contrast, severe asthma is defined by persistent symptoms despite high‐dose ICS treatment and often requires additional therapies, such as oral corticosteroids or biologic agents targeting specific inflammatory pathways.^3^, ^4^ Inconsistent use of these definitions in different clinical settings reflects the underlying heterogeneity in asthma pathophysiology and burden, which may influence treatment response.

Dupilumab is a fully human monoclonal antibody that blocks the shared receptor component for IL‐4 and IL‐13, inhibiting signaling of both IL‐4 and IL‐13, key and central drivers of type 2 inflammation.^5^, ^6^, ^7^, ^8^ In the phase 3, randomized, placebo‐controlled LIBERTY ASTHMA QUEST and VOYAGE trials, dupilumab reduced rates of severe asthma exacerbations and improved lung function and asthma control in patients with moderate‐to‐severe uncontrolled asthma aged ≥12 years in QUEST and aged 6–11 years in VOYAGE.^9^, ^10^ The benefits of dupilumab over placebo were particularly notable in patients with higher blood eosinophil and fractional exhaled nitric oxide (FeNO) levels, leading to its approval as an add‐on maintenance treatment for adults and children aged ≥6 years with severe asthma with type 2 inflammation not properly controlled by appropriate combination therapy in Europe, and moderate‐to‐severe asthma characterized by an eosinophilic phenotype or with oral corticosteroid‐dependent asthma in the US.^9^, ^10^, ^11^, ^12^ Subsequent long‐term assessment in EXCURSION, an open‐label extension study of VOYAGE, affirmed sustained efficacy of dupilumab for up to 2 years.^13^

In this post hoc study, we evaluated the efficacy of dupilumab in pediatric patients with type 2 asthma who completed the VOYAGE trial and entered the EXCURSION study, stratified by parent study baseline disease severity based on ICS dose and lung function. The aim was to evaluate dupilumab's efficacy across varying degrees of asthma severity in children.

## 2 METHODS

### 2.1 Study design

VOYAGE (NCT02948959) was a 52‐week, phase 3, randomized, double‐blind trial that included 408 children aged 6–11 years with physician‐diagnosed uncontrolled moderate‐to‐severe asthma receiving either a medium‐dose ICS in combination with a second controller or a high‐dose ICS alone or in combination with a second controller at a dose that had been stable for at least 1 month before screening. Children were randomized to receive subcutaneous dupilumab 100 or 200 mg by body weight or placebo every 2 weeks for 52 weeks.^7^ Following the completion of VOYAGE, 365 children (89.5% of the 408 enrolled in VOYAGE) subsequently entered EXCURSION (NCT03560466), an open‐label extension study in which all children received dupilumab for an additional 52 weeks.^13^ Following a protocol amendment, some children received dupilumab 300 mg every 4 weeks. Children who received placebo in VOYAGE and subsequently received dupilumab in EXCURSION comprise the placebo–dupilumab group. Those who received dupilumab in both studies comprise the dupilumab–dupilumab group.

The studies were conducted in accordance with the Declaration of Helsinki, the International Conference on Harmonisation Good Clinical Practice guideline, and applicable regulatory requirements. The local institutional review board or ethics committee at each study center oversaw trial conduct and documentation. Written informed consent was provided by parents or guardians of patients before participation in the trial.

### 2.2 Study population

This post hoc analysis included children who completed VOYAGE (placebo and combined dupilumab treatment groups) and subsequently enrolled in EXCURSION (placebo–dupilumab and dupilumab–dupilumab treatment groups) with evidence of type 2 inflammation (defined as parent study baseline blood eosinophils ≥150 cells/μL and/or FeNO ≥20 parts per billion (ppb)). For this analysis, children were stratified post hoc by asthma severity at parent study baseline based on ICS dose and lung function: those receiving medium‐dose ICS and with pre‐bronchodilator percent predicted forced expiratory volume in 1 second (FEV_1_) ≥80% were categorized as having moderate asthma; those receiving high‐dose ICS and with pre‐bronchodilator percent predicted FEV_1_ <80% were categorized as having severe asthma.^14^, ^15^ Percent predicted FEV_1_ was calculated using race‐neutral equations.

### 2.3 Endpoints

Endpoints assessed in this analysis included the unadjusted annualized rate of severe exacerbations during the 104 weeks of VOYAGE and EXCURSION and change from parent study baseline through VOYAGE and EXCURSION in pre‐bronchodilator percent predicted FEV_1_ and pre‐bronchodilator FEV_1_ *z*‐score calculated based on the race‐neutral version. According to the Global Lung Initiative, *z*‐scores values greater than −1.64 may be categorized as normal, and values between −2.51 and −4.0 reflect moderate impairments.^16^, ^17^ *z*‐scores are employed as valid measures to express the change over time in growing individuals, independent of race and ethnicity. This post hoc analysis also includes Interviewer‐Administered 7‐item Asthma Control Questionnaire (ACQ‐7‐IA) scores at the end of VOYAGE.

### 2.4 Statistical analysis

The unadjusted annualized severe exacerbations were calculated as the total number of events divided by the total number of patient‐years during the treatment period. For VOYAGE and EXCURSION, changes from parent study baseline in pre‐bronchodilator percent predicted FEV_1_ and pre‐bronchodilator FEV_1_ *z*‐scores were reported as mean (standard deviation (SD)) for both studies.

For VOYAGE, changes in ACQ‐7‐IA were reported as least squares (LS) mean (standard error (SE)) and derived using a mixed‐effect model with repeated measures (change from baseline in ACQ‐7‐IA up to Week 52 as the response variable, and treatment, age, weight group, region, baseline eosinophil level, baseline FeNO level, visit, treatment‐by‐visit interaction, baseline ACQ‐7‐IA, and baseline‐by‐visit interaction as covariates); ACQ‐7‐IA data were not collected in EXCURSION.

## 3 RESULTS

### 3.1 Study participants

A total of 179 children (49.0% of the 365 who enrolled in EXCURSION) with evidence of type 2 inflammation having completed VOYAGE and enrolled in EXCURSION met the subgroup criteria and were included in this analysis. Of these, 104 (58.1%) children met the criteria for moderate asthma: parent study baseline medium‐dose ICS and pre‐bronchodilator percent predicted FEV_1_ ≥ 80% (combined placebo, *n* = 35; combined dupilumab, *n* = 69); 75 (41.9%) children met the criteria for severe asthma: parent study baseline high‐dose ICS and pre‐bronchodilator percent predicted FEV_1_ < 80% (combined placebo, *n* = 29; combined dupilumab, *n* = 46). Patient characteristics at parent study baseline are shown by treatment group and disease severity (Table 1). Children included in this analysis were mostly male (64.8%) and White (89.4%); disease characteristics, including age at onset of asthma, number of severe asthma exacerbations experienced, pre‐ and post‐bronchodilator FEV_1_, blood eosinophil count and ACQ‐7‐IA were well balanced across treatment groups.

**TABLE 1:** Demographic and disease characteristics of children with type 2 asthma by disease severity at parent study baseline.

Characteristic	Moderate asthma		Severe asthma	
	Placebo	Dupilumab	Placebo	Dupilumab
	*n* = 35	*n* = 69	*n* = 29	*n* = 46
Age (years), mean (SD)	9.0 (1.6)	8.9 (1.5)	9.4 (1.4)	9.0 (1.7)
Male, *n* (%)	26 (74.3)	41 (59.4)	20 (69.0)	29 (63.0)
Race, *n* (%)				
White	34 (97.1)	68 (98.6)	25 (86.2)	33 (71.7)
Other	1 (2.9)	1 (1.4)	4 (13.7)	13 (28.3)
Hispanic or Latino, *n* (%)	14 (40.0)	29 (42.0)	11 (37.9)	16 (34.8)
Weight (kg), mean (SD)	38.9 (11.8)	35.0 (9.3)	37.1 (10.6)	37.2 (10.4)
Body mass index (kg/m^2^), mean (SD)	19.6 (4.4)	18.4 (3.6)	19.0 (3.4)	19.0 (3.5)
Age at onset of asthma (years), mean (SD)	4.4 (2.5)	3.5 (2.2)	3.6 (2.7)	2.4 (2.4)
Time since first diagnosis of asthma to baseline (years), mean (SD)	4.7 (2.5)	5.4 (2.6)	5.9 (2.8)	6.5 (2.6)
Number of severe asthma exacerbations experienced in the past year, mean (SD)	1.71 (1.02)	1.99 (1.40)	2.90 (2.13)	3.11 (2.87)
1, *n* (%)	20 (57.1)	32 (46.4)	6 (20.7)	15 (32.6)
2, *n* (%)	8 (22.9)	24 (34.8)	8 (27.6)	10 (21.7)
3, *n* (%)	5 (14.3)	4 (5.8)	9 (31.0)	7 (15.2)
≥4, *n* (%)	2 (5.7)	9 (13.0)	6 (20.7)	14 (30.4)
Pre‐bronchodilator FEV_1_ (L), mean (SD)	1.81 (0.43)	1.65 (0.30)	1.34 (0.36)	1.31 (0.31)
Percent predicted pre‐bronchodilator FEV_1_ (%), mean (SD)	89.91 (8.85)	86.97 (6.22)	68.10 (10.13)	67.93 (9.86)
FEV_1_ reversibility (%), mean (SD)	7.76 (9.91)	15.07 (12.01)	19.00 (11.16)	28.52 (23.64)
Post‐bronchodilator FEV_1_ (L), mean (SD)	1.95 (0.50)	1.90 (0.36)	1.60 (0.45)	1.63 (0.40)
Pre‐bronchodilator FEV_1_ *z*‐score, mean (SD)	−0.60 (0.67)	−0.80 (0.49)	−2.31 (0.67)	−2.41 (0.68)
ACQ‐7‐IA, mean (SD)	1.87 (0.70)	1.93 (0.51)	2.14 (0.73)	2.26 (0.71)
Blood eosinophil count (cells/μL), median (Q1–Q3)	380 (260–620)	470 (290–730)	520 (370–840)	630 (300–810)
<150, *n* (%)	1 (2.9)	1 (1.4)	3 (10.3)	4 (8.7)
≥150 to <300, *n* (%)	12 (34.3)	19 (27.5)	1 (3.4)	7 (15.2)
≥300, *n* (%)	22 (62.9)	49 (71.0)	25 (86.2)	35 (76.1)
Total IgE (IU/mL), median (Q1–Q3)	403 (215–721)	488 (158–1309)	386 (71–911)	490 (231–868)
FeNO (ppb), median (Q1–Q3)	21 (13–30)	19 (11–34)	29 (14–45)	26 (12–50)
<20, *n* (%)	16 (45.7)	34 (49.3)	10 (34.5)	16 (34.8)
≥20 to <35, *n* (%)	14 (40.0)	16 (23.2)	9 (31.0)	11 (23.9)
≥35, *n* (%)	4 (11.4)	16 (23.2)	9 (31.0)	18 (39.1)
Missing, *n* (%)	1 (2.9)	3 (4.3)	1 (3.4)	1 (2.2)
TARC (pg/mL), median (Q1–Q3)	432 (316–595)	397 (263–592)	348 (216–713)	414 (332–624)

Abbreviations: ACQ‐7‐IA, Interviewer‐Administered 7‐item Asthma Control Questionnaire; FeNO, fractional exhaled nitric oxide; FEV_1_, forced expiratory volume in 1 second; IgE, immunoglobulin E; IU, International Units; ppb, parts per billion; Q, quartile; SD, standard deviation; TARC, thymus‐ and activation‐regulated chemokine.

By the definitions of moderate and severe asthma used in this study, children with severe asthma also had lower lung function measures than those with moderate asthma (mean (SD) pre‐bronchodilator percent predicted FEV_1_ = 68.10% (10.13) for those on placebo versus 67.93% (9.86) for those on dupilumab, compared to 89.91% (8.85) for those on placebo versus 86.97% (6.22) for those on dupilumab) and higher FEV_1_ reversibility (mean (SD) 19.00% (11.16) for those on placebo versus 28.52% (23.64) for those on dupilumab, compared to 7.76% (9.91) for those on placebo versus 15.07% (12.01) for those on dupilumab). Children with moderate asthma had normal pre‐bronchodilator FEV_1_ *z*‐scores at baseline (mean *z*‐score: greater than −1.64), while baseline pre‐bronchodilator FEV_1_ *z*‐scores for those with severe asthma indicated moderate impairment (mean *z*‐score: −2.31 to −2.41).

Children with severe asthma had worse asthma control than those with moderate asthma, as indicated by higher ACQ‐7‐IA scores at parent study baseline. Markers of type 2 inflammation (blood eosinophil counts and FeNO levels) were higher in children with severe asthma; more children with severe asthma had a blood eosinophil count ≥300 cells/μL and FeNO ≥20 ppb at parent study baseline than those with moderate asthma.

### 3.2 Annualized rate of severe asthma exacerbations

**FIGURE 1:**
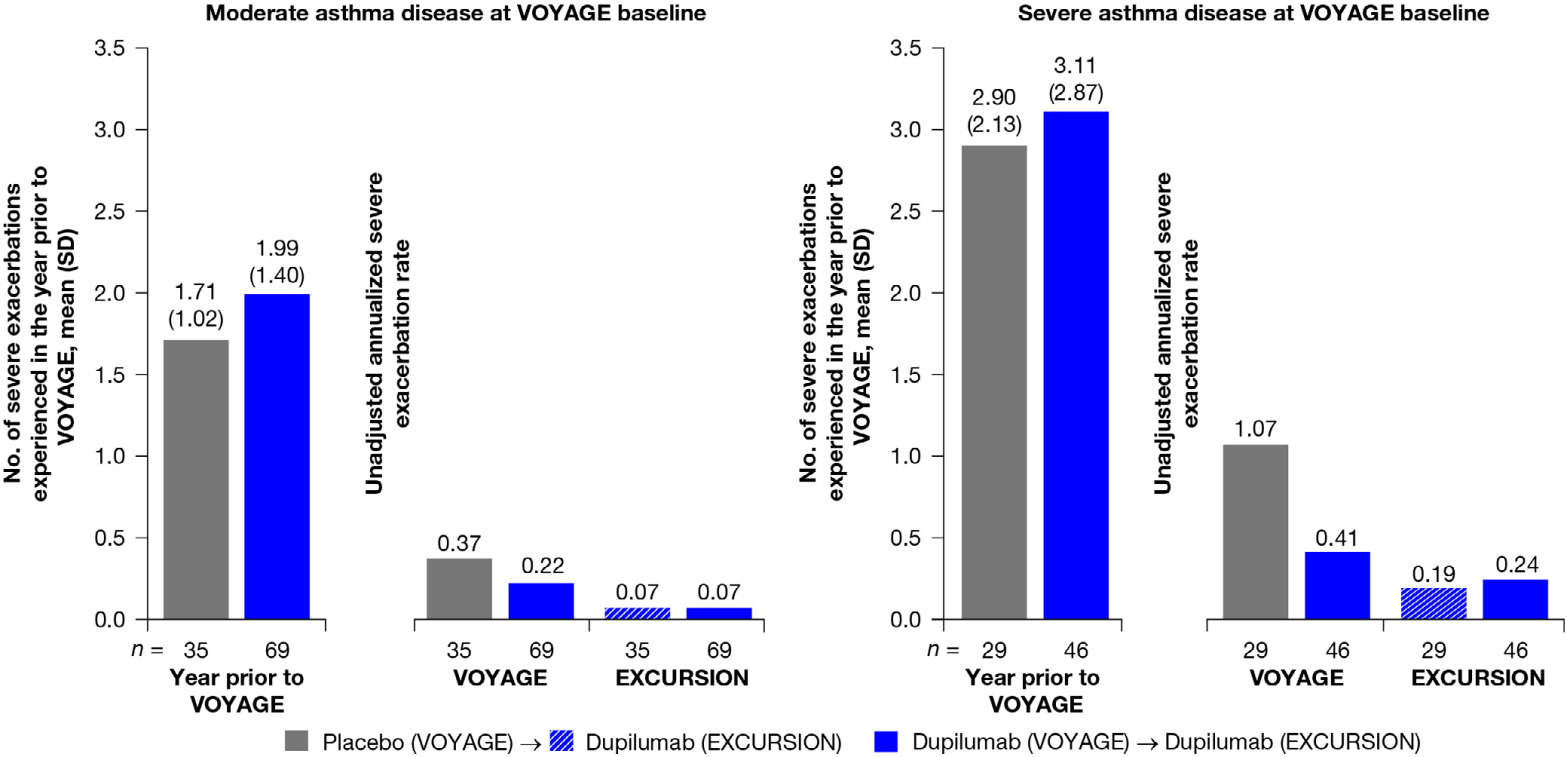
Unadjusted annualized rates of severe asthma exacerbations during VOYAGE and EXCURSION in children with type 2 asthma by disease severity. SD, standard deviation.

Children with severe asthma experienced more exacerbations in the year prior to VOYAGE, with a mean (SD) of 3.11 (2.87) for those on dupilumab versus 2.90 (2.13) for those on placebo, compared with those with moderate asthma, with a mean (SD) of 1.99 (1.40) for those on dupilumab versus 1.71 (1.02) for those on placebo. At Week 52 of VOYAGE, dupilumab compared to placebo reduced unadjusted annualized severe asthma exacerbation rates to 0.22 versus 0.37 in children with moderate asthma (15 events/69.0 patient‐years versus 13 events/35.1 patient‐years, respectively), and 0.41 versus 1.07 in children with severe asthma (19 events/46.1 patient‐years versus 31 events/29.1 patient‐years, respectively). At Week 52 of EXCURSION, reductions in severe exacerbation rates were sustained in the dupilumab–dupilumab group and achieved in the placebo–dupilumab group, regardless of asthma severity. In children with moderate asthma, the unadjusted exacerbation rate was 0.07 in both treatment groups (four events/61.3 patient‐years for the dupilumab–dupilumab group and two events/30.9 patient‐years for the placebo–dupilumab group). In children with severe asthma, rates were 0.24 (eight events/33.7 patient‐years) for the dupilumab–dupilumab group and 0.19 (four events/21.4 patient‐years) for the placebo–dupilumab group (Figure 1).

### 3.3 Change in pre‐bronchodilator percent predicted FEV_1_

**FIGURE 2:**
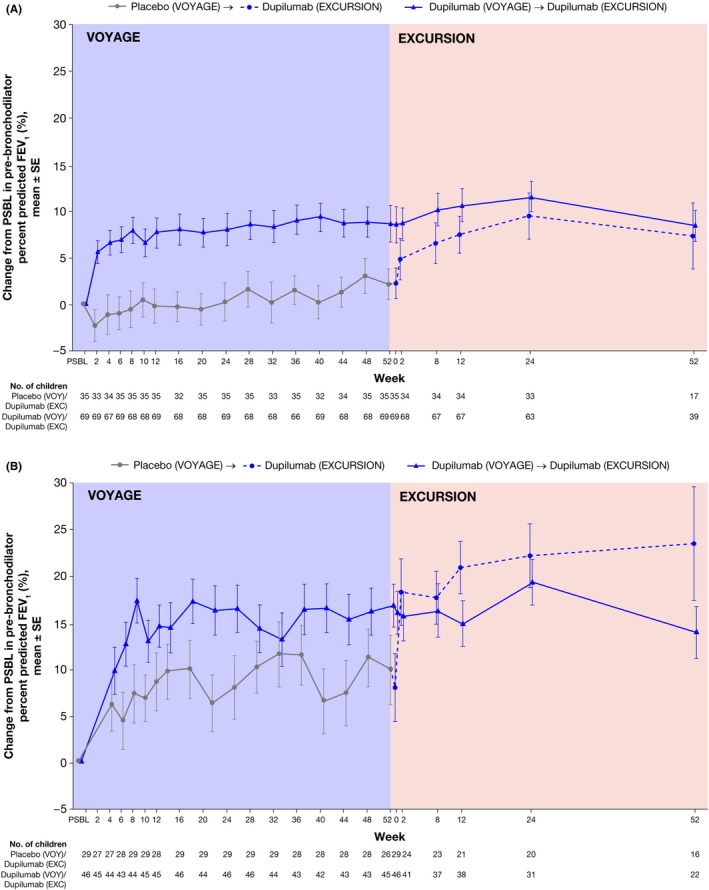
Change from parent study baseline in pre‐bronchodilator percent predicted FEV_1_ during VOYAGE and EXCURSION treatment periods in patients with type 2 moderate (A) and severe (B) asthma disease at VOYAGE baseline. EXC, EXCURSION; FEV_1_, forced expiratory volume in 1 second; PSBL, parent study baseline; SE, standard error; VOY, VOYAGE.

Change from parent study baseline in pre‐bronchodilator percent predicted FEV_1_ during VOYAGE and EXCURSION is reported by treatment group and asthma severity (Figure 2). At Week 52 of VOYAGE, the mean (SD) change from parent study baseline in pre‐bronchodilator percent predicted FEV_1_ was higher in children who received dupilumab compared to placebo, with improvements of 8.6 (16.3) percentage points versus 2.1 (9.7) percentage points in those with moderate asthma (Figure 2A) and 16.7 (15.5) percentage points versus 9.8 (19.0) percentage points in patients with severe asthma (Figure 2B). These improvements were sustained through Week 52 of EXCURSION in the dupilumab–dupilumab group and achieved in the placebo–dupilumab group, with improvements of 8.4 (10.4) percentage points and 7.3 (14.6) percentage points in children with moderate asthma, and 14.00 (13.01) percentage points and 23.5 (24.3) percentage points in children with severe asthma.

### 3.4 Change in pre‐bronchodilator FEV_1_ *z*‐score

**FIGURE 3:**
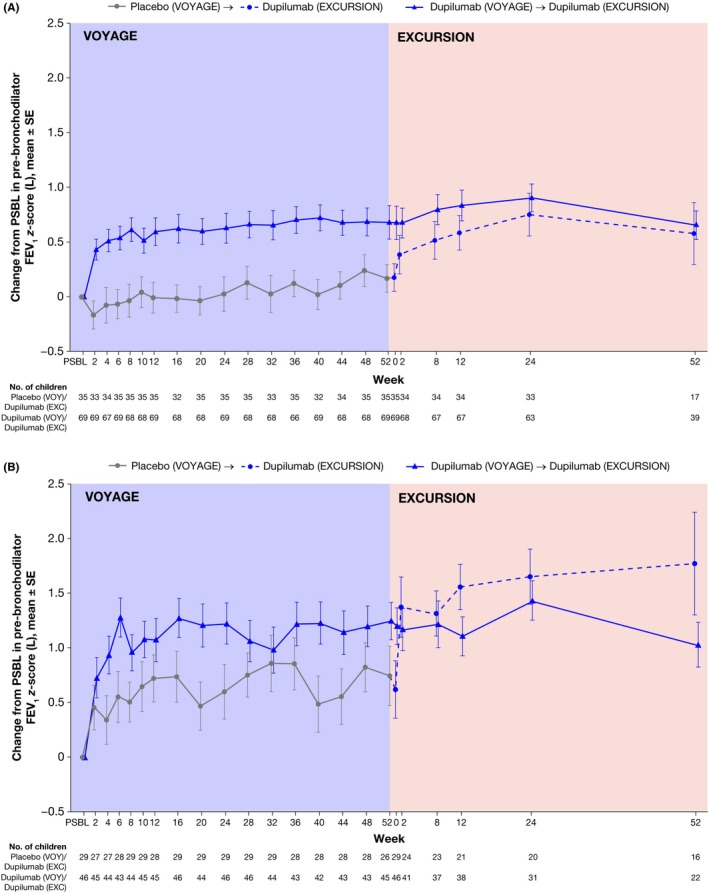
Change from parent study baseline in pre‐bronchodilator FEV_1_ *z*‐score during VOYAGE^a^ and EXCURSION^b^ treatment periods in patients with type 2 moderate (A) and severe (B) asthma disease at VOYAGE baseline. EXC, EXCURSION; FEV_1_, forced expiratory volume in 1 second; PSBL, parent study baseline; SE, standard error; VOY, VOYAGE. ^a^Height was measured at the screening and randomization visits and every subsequent visit. Any missing heights utilized for *z*‐score calculations used height data from the previous available visit. ^b^Height was only measured at the enrollment visit into EXCURSION and at the end of treatment visit. Heights used for *z*‐score calculations used height data from the previous available visit.

At parent study baseline, by definition, 100.0% of children with moderate asthma had normal pre‐bronchodilator FEV_1_ *z*‐scores compared with 3.5% of children with severe asthma who received placebo versus 10.9% who received dupilumab. At Week 52 of VOYAGE, treatment with dupilumab led to greater improvements in mean (SD) pre‐bronchodilator FEV_1_ *z*‐scores compared to placebo in patients with both moderate (0.68 (1.27) vs. 0.17 (0.75)) and severe asthma (1.25 (1.14) vs. 0.75 (1.37)) (Figure 3A,B). Improvements in pre‐bronchodilator FEV_1_ *z*‐scores were sustained through Week 52 of EXCURSION in the dupilumab–dupilumab group, with a mean (SD) change from parent study baseline of 0.65 (0.81) and 1.03 (0.96) with moderate and severe asthma, respectively, and achieved in the placebo–dupilumab group, with a mean (SD) change of 0.58 (1.17) and 1.77 (1.88) with moderate and severe asthma, respectively (Figure 3A,B).

### 3.5 Change in ACQ‐7‐IA scores

At parent study baseline, ACQ‐7‐IA scores were higher, indicating worse asthma control, in children with severe asthma compared to those with moderate asthma (mean (SD) 2.14 (0.73) for those on placebo versus 2.26 (0.71) for those on dupilumab, compared to 1.87 (0.70) for those on placebo versus 1.93 (0.51) for those on dupilumab). Statistically significant improvements in ACQ‐7‐IA scores were observed as early as Week 12 of VOYAGE, and sustained to the end of the study period, in children with severe asthma, and were observed in children with moderate asthma by Week 52. At Week 52 of VOYAGE, the LS mean difference (95% confidence interval (CI)) in ACQ‐7‐IA scores for dupilumab versus placebo was −0.29 (95% CI −0.57 to −0.02; *p* = .0387) in children with moderate asthma and −0.56 (95% CI −0.83 to −0.29; *p* = .0001) in children with severe asthma (Table 2).

**TABLE 2:** Change from baseline in ACQ‐7‐IA score in children with type 2 asthma by disease severity during the VOYAGE^a^ treatment period.

ACQ‐7‐IA score	Moderate asthma		Severe asthma	
	Placebo	Dupilumab	Placebo	Dupilumab
	*n* = 35	*n* = 69	*n* = 29	*n* = 46
Baseline, mean (SD)	1.87 (0.70)	1.93 (0.51)	2.14 (0.73)	2.26 (0.71)
Change from baseline, Week 2	*n* = 34	*n* = 66	*n* = 28	*n* = 45
LS mean (SE)^b^	−0.47 (0.14)	−0.77 (0.10)	−0.56 (0.18)	−0.57 (0.14)
LS mean diff vs. placebo (95% CI)^b^		−0.29 (−0.60 to 0.01)		−0.01 (−0.44 to 0.41)
*p*‐value vs. placebo^b^		0.0619		0.9526
Change from baseline, Week 12	*n* = 34	*n* = 66	*n* = 28	*n* = 45
LS mean (SE)^b^	−1.00 (0.13)	−1.12 (0.10)	−0.72 (0.14)	−1.25 (0.12)
LS mean diff vs. placebo (95% CI)^b^		−0.12 (−0.42 to 0.18)		−0.53 (−0.87 to −0.20)
*p*‐value vs. placebo^b^		0.4342		0.0022
Change from baseline, Week 24	*n* = 34	*n* = 66	*n* = 28	*n* = 45
LS mean (SE)^b^	−1.10 (0.12)	−1.25 (0.08)	−1.05 (0.13)	−1.36 (0.11)
LS mean diff vs. placebo (95% CI)^b^		−0.15 (−0.40 to 0.11)		−0.32 (−0.62 to −0.01)
*p*‐value vs. placebo^b^		0.2488		0.0407
Change from baseline, Week 52	*n* = 34	*n* = 66	*n* = 28	*n* = 45
LS mean (SE)^b^	−1.09 (0.13)	−1.38 (0.09)	−1.02 (0.12)	−1.57 (0.10)
LS mean diff vs. placebo (95% CI)^b^		−0.29 (−0.57 to −0.02)		−0.56 (−0.83 to −0.29)
*p*‐value vs. placebo^b^		0.0387		0.0001

Abbreviations: ACQ‐7‐IA, Interviewer‐Administered 7‐item Asthma Control Questionnaire; CI, confidence interval; FeNO, fractional exhaled nitric oxide; LS, least squares; MMRM, mixed‐effect model with repeated measures; SD, standard deviation; SE, standard error.

^a^ ACQ‐7‐IA data were not collected in EXCURSION.

^b^ Derived from MMRM with change from baseline in ACQ‐7‐IA up to Week 52 as the response variable, and treatment, age, weight group, region, baseline eosinophil level, baseline FeNO level, visit, treatment‐by‐visit interaction, baseline ACQ‐7‐IA, and baseline‐by‐visit interaction as covariates.

## 4 DISCUSSION

This post hoc analysis evaluated dupilumab efficacy during the VOYAGE and EXCURSION studies in children with type 2 asthma stratified by their disease severity at parent study baseline. Results showed that during VOYAGE, dupilumab reduced severe exacerbation rates and improved lung function and asthma control in children aged 6–11 years with type 2 asthma, irrespective of parent study baseline disease severity defined by lung function and ICS dose. Improvements with dupilumab treatment during VOYAGE were maintained for up to 2 years in EXCURSION and achieved in those who switched from placebo to dupilumab, regardless of disease severity at parent study baseline.

These findings expand published results from the VOYAGE and EXCURSION studies by demonstrating consistent efficacy of dupilumab across clinically defined severity subgroups based on ICS dose and lung function. While improvements in key clinical outcomes were observed regardless of asthma severity at baseline, the effects of dupilumab were numerically greater in children with severe asthma compared to those with moderate asthma, particularly in heightened improvements in lung function. However, these children exhibited a greater degree of lung function impairment at baseline, more frequent exacerbations in the year prior to VOYAGE, and worse asthma control, suggesting more room for improvement with dupilumab treatment.

Nonetheless, these results reinforce the relevance of targeting underlying inflammation, particularly in children whose moderate or severe asthma remains uncontrolled despite standard of care inhaler therapies. Our results suggest that dupilumab acts on pathobiological mechanisms in severe asthma beyond those addressed by ICS specifically by blocking IL‐4 and IL‐13, cytokines that are broadly involved in type 2 inflammatory processes such as production and proliferation of Th2 cells, B cell class switching to immunoglobulin E (IgE) production, eosinophil and mast cell airway infiltration, airway fibrosis and remodeling, and mucus hypersecretion.^18^, ^19^, ^20^ By reducing dependence on oral corticosteroids or high‐dose ICS, dupilumab may help minimize long‐term risks such as hypertension and obesity, growth impairment, immunosuppression, and adrenal insufficiency due to suppression of the hypothalamic–pituitary–adrenal axis.^21^, ^22^, ^23^

This analysis has several limitations. Participation in EXCURSION was voluntary and limited to children who completed VOYAGE, potentially introducing selection bias. This setup may also introduce a treatment bias, as children who responded positively to dupilumab in VOYAGE might have been more likely to continue compared with those who received placebo. Additionally, the study had a disproportionate representation of White, male children, which may also limit its generalizability. The single‐arm, open‐label design of the EXCURSION study also introduces potential limitations to data analysis and interpretation. Therefore, careful consideration is necessary when interpreting efficacy outcomes from this study.

Overall, these findings support dupilumab as a treatment option in children with moderate‐to‐severe type 2 asthma, providing sustained reductions in exacerbations and improvements in lung function and symptom control across varying levels of disease severity. The findings highlight the potential of targeted biologic therapies in managing difficult‐to‐treat pediatric asthma.

## 5 CONCLUSION

In children aged 6–11 years with uncontrolled, moderate‐to‐severe, type 2 asthma, dupilumab was efficacious in reducing exacerbations and improving lung function and asthma control during a 2‐year treatment period across varying degrees of asthma severity, with numerically heightened efficacy in children with severe disease.

## AUTHOR CONTRIBUTIONS

**Daniel J. Jackson:** Conceptualization; writing – original draft; writing – review and editing. **Monika Gappa:** Conceptualization; writing – original draft; writing – review and editing. **Leonard B. Bacharier:** Conceptualization; writing – original draft; writing – review and editing; investigation. **Antoine Deschildre:** Conceptualization; writing – original draft; writing – review and editing. **Kristie Ross:** Conceptualization; writing – original draft; writing – review and editing. **Changming Xia:** Methodology; software; validation; formal analysis; data curation; writing – original draft; writing – review and editing. **Olivier Ledanois:** Conceptualization; resources; writing – original draft; writing – review and editing; supervision; project administration. **Katherine Miller:** Conceptualization; resources; writing – original draft; writing – review and editing; supervision; project administration.

## FUNDING INFORMATION

Research sponsored by Sanofi and Regeneron Pharmaceuticals Inc. ClinicalTrials.gov Identifiers: NCT02948959 and NCT03560466.

## CONFLICT OF INTEREST STATEMENT

**D.J. Jackson** has been a consultant for Areteia, AstraZeneca, Avillion, Genentech, GSK, Regeneron Pharmaceuticals Inc., and Sanofi; has participated in data and safety monitoring board for AstraZeneca, Pfizer, and Upstream Bio; has received research grant funding from GSK, OM Pharma, and Regeneron Pharmaceuticals Inc. **M**. **Gappa** has received speaker/consultant fees from ALK, AstraZeneca, DBV, Nestlé, Regeneron Pharmaceuticals Inc., and Sanofi. **L.B**. **Bacharier** has received speaker/advisory fees from AstraZeneca, GSK, Regeneron Pharmaceuticals Inc., and Sanofi; has received research support from OM Pharma; has participated in data and safety monitoring board for AstraZeneca, Cystic Fibrosis Foundation, and DBV Technologies; has received research support from NIH, Sanofi, and Vectura. **A**. **Deschildre** has received speaker/consulting fees from Aimmune Therapeutics, ALK, AstraZeneca, Celltrion DBV Technologies, GSK, Nestlé, Regeneron Pharmaceuticals Inc., Sanofi, Stallergenes Greer, and Viatris. **K**. **Ross** has received speaker/consultant fees from AstraZeneca, Boehringer Ingelheim, and GSK; has received research support from AstraZeneca, Boehringer Ingelheim, and Sanofi. **C**. **Xia** and **K. Miller** are employees and shareholders of Regeneron Pharmaceuticals Inc. **O**. **Ledanois** is an employee at Sanofi and may hold stock and/or stock options in the company.

## Data Availability

Qualified researchers may request access to study documents (including the clinical study report, study protocol with any amendments, blank case report form, statistical analysis plan) that support the methods and findings reported in this manuscript. Individual anonymized participant data will be considered for sharing once the product and indication have been approved by major health authorities (e.g., FDA, EMA, PMDA, etc), if there is legal authority to share the data and there is not a reasonable likelihood of participant re‐identification. Submit requests to https://vivli.org/.
